# Experimental and Finite Element Analysis of the Tensile Behavior of Architectured Cu-Al Composite Wires

**DOI:** 10.3390/ma14216305

**Published:** 2021-10-22

**Authors:** Alireza Dashti, Clément Keller, Benoit Vieille, Alain Guillet, Christophe Bouvet

**Affiliations:** 1 Groupe de Physique des Matériaux, UMR CNRS 6634, Normandie Université, Avenue de l’Université, 76800 Saint-Étienne-du-Rouvray, France; benoit.vieille@insa-rouen.fr (B.V.); alain.guillet@insa-rouen.fr (A.G.); 2 LGP—Laboratoire Génie de Production, ENIT—École Nationale d’Ingénieurs de Tarbes, 47 Av. d’Azereix, 65000 Tarbes, France; ckeller@enit.fr; 3 IGMT, LMS Supaéro, BP 54032, CEDEX 4, 31055 Toulouse, France; christophe.bouvet@isae-supaero.fr

**Keywords:** wire drawing, Cu-Al composite wires, finite element analysis

## Abstract

The present study investigates, experimentally and numerically, the tensile behavior of copper-clad aluminum composite wires. Two fiber-matrix configurations, the conventional Al-core/Cu-case and a so-called architectured wire with a continuous copper network across the cross-section, were considered. Two different fiber arrangements with 61 or 22 aluminum fibers were employed for the architectured samples. Experimentally, tensile tests on the two types of composites show that the flow stress of architectured configurations is markedly higher than that of the linear rule of mixtures’ prediction. Transverse stress components and processing-induced residual stresses are then studied via numerical simulations to assess their potential effect on this enhanced strength. A set of elastic-domain and elastoplastic simulations were performed to account for the influence of Young’s modulus and volume fraction of each phase on the magnitude of transverse stresses and how theses stresses contribute to the axial stress-strain behavior. Besides, residual stress fields of different magnitude with literature-based distributions expected for cold-drawn wires were defined. The findings suggest that the improved yield strength of architectured Cu-Al wires cannot be attributed to the weak transverse stresses developed during tensile testing, while there are compelling implications regarding the strengthening effect originating from the residual stress profile. Finally, the results are discussed and concluded with a focus on the role of architecture and residual stresses.

## 1. Introduction

Abundant copper demand for electrical applications from various sectors has prompted manufacturers to reduce material costs by replacing this rather expensive and high-density metal partly or entirely. Lower-density and more affordable aluminum-copper (Al-Cu) composite wire is an example of such efforts. The following paragraphs provide a summary of the different features of Al-Cu wires and several other similar composite systems (developed by various techniques) already investigated. The missing aspects and the property of interest to be researched in the current work are then presented at the end of this section. Among those already-studied features are the investigations covering the mechanical behavior and finite element modelling of the manufacture processes of severely cold worked composite systems akin to the one under study in this work. Khosravifard and Ebrahimi [1] investigated the parameters affecting the interface strength of extruded Al/Cu clad bimetal rods along with FEM analysis of the extrusion process. Feng et al. [2] examined the compressive mechanical behavior of Al/Mg composite rods with different types of Al sleeve.

Gu et al. [3] modelled the elastic behavior of architectured and nanostructured Cu–Nb composite wires produced by accumulative drawing and bundling (a severe plastic deformation technique) in a multiscale manner. Priel et al. [4] did a computational study (validated by experiments) on co-extrusion of an Mg/Al composite billet and suggested a set-up named “Floating Core” as being ideal.

Knezevic et al. [5] made a comparison between three die designs with a material-based approach towards the extrusion of bimetallic tubes discussing the criteria that are to be met for proper solid-state bonding.

Moreover, a great deal of research has been done addressing the mechanical behavior of metallic and non-metallic fiber-reinforced composites. Ochiai [6] performed an extensive study on the effect of interface on deformation and fracture behavior of metallic matrix fiber-reinforced composites. Kelly and Lilholt [7] researched stress-strain curve of a fiber-reinforced composite of tungsten wires embedded in a pure copper matrix. Kelly and Tyson [8] studied tensile properties of metallic fiber-reinforced composite systems of copper/tungsten and copper-molybdenum. Ebert et al. [9] analyzed the stress-strain behavior of concentric composite cylinders. Sapanathan et al. [10] spiral extruded an aluminum/copper composite to study its bond strength and interfacial characteristics. Hao et al. [11] developed a novel multifunctional NiTi/Ag hierarchical composite, inspired by the hierarchical design of the tendon, by repeated assembling and wire drawing. Tyson and Davies [12] investigated the shear stresses associated with stress transfer during fiber reinforcement with the help of photoelasticity. Superconducting materials embedded into a copper matrix as multifilaments [13] and aluminum-steel fiber composites [14] are the other systems with similarities to the Al-Cu composites under investigation in the current study.

The conventional copper-clad aluminum wire (CCA or single-Al-fiber Al-Cu composite wire) is currently being widely used in the electrical industry [15]. Architectured copper-clad aluminum wire (ACCA or multi-Al-fiber Al-Cu composite wire), however, has proved to be superior in a variety of areas offering improved thermal diffusivity [16] and proper electrical conductivity at both low and high frequencies. Moreover, in a previous article, the authors have reported that ACCA samples exhibit rather complex mechanical behavior in both as-drawn and heat-treated states (see [17] for more details).

The novelty of this work is the investigation of the origin of the understudied mechanical behavior of the novel architectured Cu-Al composite wires and its promising implications in terms of the in-service reliability. The objective of this article is then to better understand the mechanical behavior of Cu-Al wires with different fiber-matrix configurations. Along with the conventional CCA wire, two architectured configurations (ACCA) with different numbers of Al fibers were investigated. A first assessment of the mechanical properties based on the experimental tensile curves is proposed, revealing improved flow stress for architectured configurations.

Numerical simulations of CCA and ACCA configurations were then performed to find the impact of fiber-matrix configurations on the axial stress-strain behavior of these materials. Particularly, the influence of I- transverse interactions and II- processing-induced residual stresses on the mechanical behavior were investigated. The use of finite element analysis is necessary when dealing with the mechanical behaviors that are not easy to understand and interpret experimentally. Crack and fracture behavior are instances of such studies [18,19]. A great complexity in the current work is the experimental measurement of radial and circumferential stresses developing at the interface of the fine Al fibers (tens of micron wide) and the Cu matrix during tensile testing of Arcitectured and even conventional Cu-Al wires. The results show that the processing-induced residual stresses most probably explain the exceptional mechanical properties of architectured wires.

## 2. Material and Experimental Procedure

**Figure 1:**
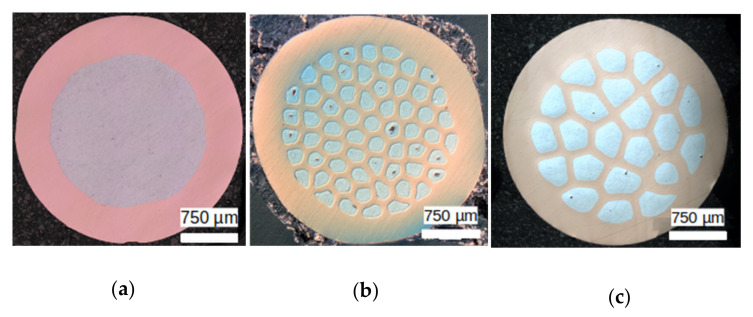
Illustration of the cross-section of the the different 3 mm diameter wires considered in this study: (**a**) conventional CCA; (**b**) architectured wire with 61 aluminum fibers and (**c**) architectured wire with 22 aluminum fibers.

Copper clad aluminum wires are produced by cold-drawing. For all wires, fully annealed high purity Oxygen Free High Conductivity (OFHC) copper and 99.5% pure Al were employed. For the fabrication of CCA, a copper tube of an outer diameter of 12 mm and inner diameter of 8 mm and an approximately 8 mm-aluminum rod were simultaneously cold-drawn down to 3 mm. For the ACCA drawing, CCA wires were restacked in a copper tube and were further cold-drawn. For these specific architectured wires, two configurations were manufactured, one with 61 restacked 1 mm-CCA wires (labelled ACCA_61_) and a second one with 22 restacked 1.7 mm-wires (labelled ACCA_22_). All wires were cold-drawn down to 3 mm without inter-operational heat-treatments. For the CCA wires, the aluminum volume fraction is about 50% whereas values of 25% and 32% are associated to the ACCA_61_ and ACCA_22_, respectively. Details about the manufacturing process can be found in the two previous articles [15,17]. The corresponding cross-sections of the three microstructures, imaged via optical microscopy, are illustrated in Figure 1.

Simulation of the CCA and ACCA behavior under tensile loading requires the stress-strain data of each component (Al and Cu). For that reason, as-drawn samples of both pure Cu and Al with the aforementioned compositions (threes samples each) were prepared for tensile testing to provide the FEA software with the required input. To prepare the above tensile test samples, an aluminum rod and a copper rod of the same initial diameter of 8 mm, heat-treated for three hours at 300 °C and 500 °C respectively, were cold drawn down to 2 mm each. This was to have the same amount of plastic deformation undergone by a 3 mm-CCA composite wire (considered for simulations) stored in pure Al and Cu samples.

The final diameter of the rods was obtained from the following relation for calculating the drawing strain:

(1) $$\eta =2\ln\frac{D_{0}}{D}$$

where D_0_ and D are the initial and final diameters respectively. An MTS Criterion Model 43 10 kN-universal testing machine (MTS, Eden Prairie, MN, USA) was used to perform displacement-controlled tensile tests at room temperature and the strain was measured via a conventional 25 mm-gage length extensometer. Samples were mounted on specialized wire tensile testing grips to minimize stress concentration and were strained at an initial strain rate of 0.004 s^−1^ to avoid possible viscous effects.

**Figure 2:**
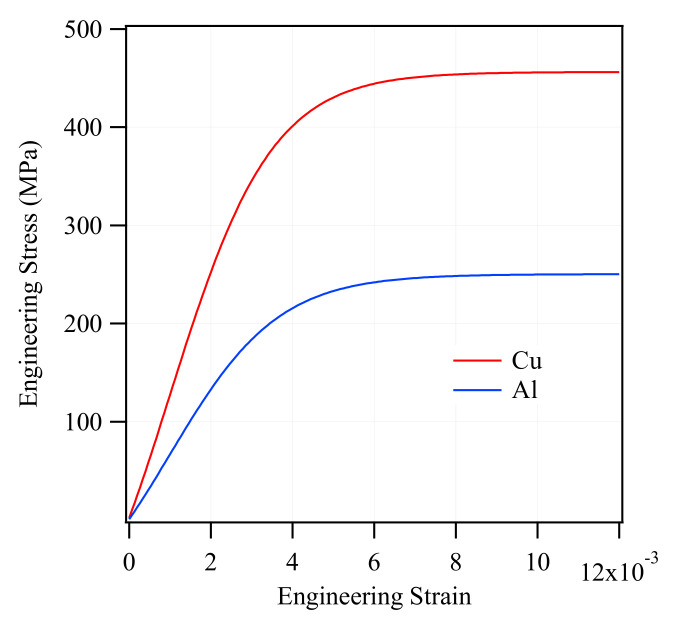
Experimental engineering stress-strain curves of pure copper and aluminum (as-drawn).

Engineering stress-strain curves of experimentally tensile-tested pure Al and Cu are plotted in Figure 2. The following curves were then converted into true stress-strain curves and were used as input for elastoplastic simulation of CCA and ACCA wires.

## 3. Numerical Procedure

A comprehensive explanation of the simulation approach is presented in the first subsection. The second subsection is devoted to the simulation details.

### 3.1. Parameters and Methodology

The application of finite element method made it possible to effectively study various parameters involved from a behavioral perspective. Assumptions such as perfect fiber-matrix interface and isotropic behavior were made for the sake of simplicity. As mentioned earlier, the two key factors I- transverse stresses and II- residual stresses (RS) were investigated in a set of numerical simulations. To this end, evolution of transverse (radial and circumferential) stress components under a tensile load was modelled in both elastic and elastoplastic domains for CCA samples.

Tensile elastic-plastic behavior of an ACCA model, created from an actual microstructure, was also studied to discover the potentially distinct development of lateral stresses in this novel configuration. For the sake of conciseness, only the ACCA_61_ configuration was considered for simulation. The effect of predefined fields of residual stress in both CCA and ACCA wires was also studied independently. The idea was to realize how significant the contribution of lateral and residual stresses could be to the axial stress-strain behavior of these bimetallic composites separately. CCA simulations hold clues to understanding the more complex tensile behavior of the architectured samples (ACCAs).

#### 3.1.1. Transverse Stresses

##### CCA Elastic Simulations

There are complexities associated with the elastoplastic behavior of these materials, originating from the formation of yield fronts and gradual elastic-to-plastic transition [9]. In a first attempt to avoid those intricacies, a number of elastic-domain CCA wire simulations were independently run, with the major parameters involved in the evolution of transverse stresses taken into consideration. Those parameters include Young’s modulus and Al/Cu volume fraction.

Therefore, two 10-mm-long CCA samples of the same outer diameter of 3 mm (arbitrary dimensions), containing an aluminum core and a copper case were modelled. One of the two samples contains 75% Al (2.6 mm-Al core) and the other 25% Al (1.5 mm-Al core). The volume fraction of the experimental CCA wire lies in between these two values. This was to account for the role of volume fraction when one of the phases prevails.

It is known from the literature that the elastic behavior of pure copper is largely anisotropic. Its Young’s modulus depends on the texture developments and can range between 60 and 200 GPa as plotted and discussed by Pal-Val et al. [20] for different crystallographic directions. For that reason, three different Young’s modulus values of 60, 170 and 200 GPa, corresponding to the dominance of [001], [011] and [111] orientations in the order given, were chosen to take account of Young’s modulus effect. Unlike Cu, the elastic behavior of Al is almost isotropic and the Young’s modulus alterations of pure aluminum and many aluminum alloys, following cold working and heat-treatment, vary slightly by 10 percent at most [21]. Hereby, an average value of 70 GPa was considered for Al. The elastic-domain impact of Poisson’s ratio value difference between the components of bimetallic fiber-composites is generally trivial [9]. In summary, three Young’s modulus values for Cu and 2 volume fractions were opted for a total number of 6 simulations. The CCA elastic simulations are summarized in Table 1.

##### CCA and ACCA Elastoplastic Simulations

It is known that radial and circumferential stresses may become more important in terms of contribution to the axial stress-strain behavior as one of the two phases in a bimetallic cylindrical composite plasticizes first and the other remains elastic within a certain strain range. This is because the already-yielded component could be assumed to have a Poisson’s ratio of 0.5 (due to the incompressible nature of plasticity) and the other would still possess the elastic-domain Poisson’s ratio value. Therefore, the difference between the values of Poisson’s ratio of the two materials would become greater for a certain range of strain before the elastic component begins to behave plastically as well [9].

Hereby, numerical tensile testing of a set of 3 mm-diameter CCA wires (actual dimension) of four different Al/Cu volume fractions was opted to be modelled with elastic-plastic behavior (without accounting for the Al/Cu interface and residual stresses). Four volume fractions were chosen to have a statistically better approximation of the order of magnitude of transverse stresses. The goal was to discover the degree to which lateral stresses, alone, can possibly influence the tensile behavior. Table 2 lists all the CCA elastoplastic simulations performed.

**Figure 3:**
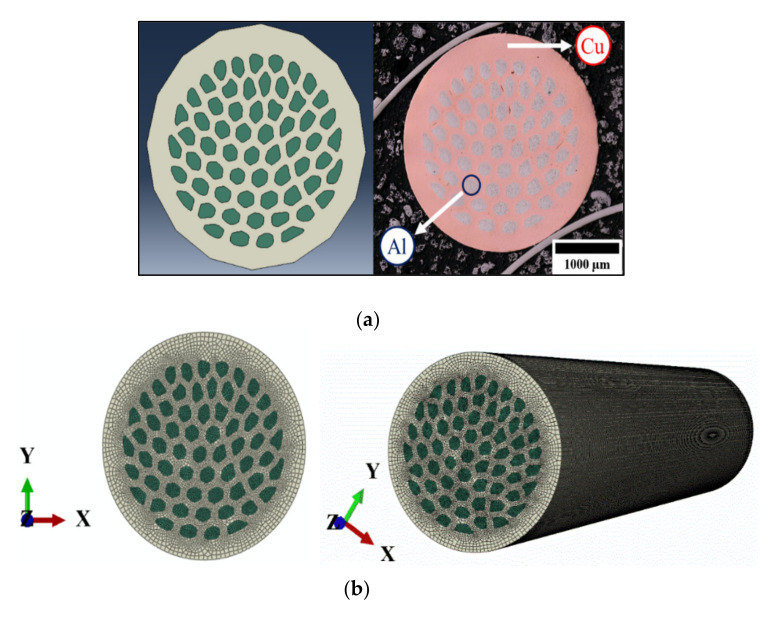
≈25%Al-ACCA sample and corresponding finite element models (**a**) actual microstructure and corresponding FEA model (**b**) meshed ACCA wire model.

Having insights provided from the elastoplastic simulation of CCA wires, a 3-mm diameter ACCA_61_ wire was modelled from the actual microstructure of its transversal cross section (see Figure 3a). The wire contains 61 Al fibers (about 25 percent of the total volume fraction) embedded in a Cu matrix. The corresponding finite element models are presented in Figure 3a,b.

#### 3.1.2. Residual Stresses

Mechanical residual stresses built up during cold drawing of metals are known to come from the non-uniform nature of plastic deformation in this process. There is a qualitative feature from the literature based on which residual stress simulations are argued in this paper. This feature is the formation of a rather wide range of compressive residual stresses in the central part of a cold-drawn bar and a narrower range of tensile residual stress in its outer part, away from the center. Axial tensile residual stresses forming near the wire surface have detrimental effects on the tensile strength of drawn wires. Modifying the residual profile through the wire cross section by reducing those stresses and boosting the formation of compressive residual stresses favors the yield strength [22]. Atienza and Elices [23] suggest such RS distribution for cold drawn steel wires investigated both numerically and experimentally. Ripoll et al. [24] report a similar RS distribution pattern in their investigation of tungsten wires. Bullough and Hartley [25] introduce an analytical model for co-deformed Cu-Al rods confirming the above-mentioned RS distribution.

Consistent with the literature on the magnitude and distribution pattern of drawing-induced residual stresses, a behavioral assessment was conducted. One objective was to analyze how the distinct fiber-matrix configuration of an ACCA sample can possibly affect the axial stress-strain behavior of Al-Cu composite wires of the same Al/Cu volume fraction but different architecture.

For the sake of simplicity, this comparison was made irrespective of the fact that ACCA is more strained than CCA and more compressive residual stresses are expected to form in architectured wires. Hereby, the aforementioned 3-mm ACCA sample containing about 25% Al and its corresponding 3-mm CCA sample with 25% Al were considered.

**Figure 4:**
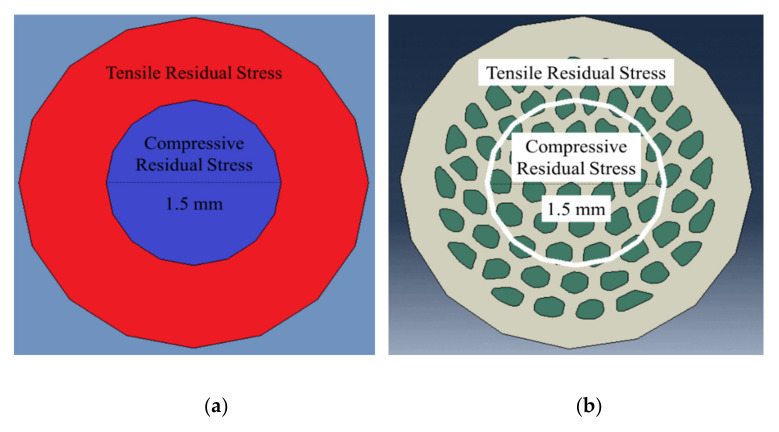
Predefined fields of residual stresses in (**a**) CCA and (**b**) ACCA wires (transversal cross-section).

Same-diameter cylinders of compressive residual stress were defined in the center of both wires with hollow cylinders of the same width under tensile residual stress, as illustrated in Figure 4a,b. Residual stress modelling and analyses were based on the values reported for copper-clad aluminum wires fabricated by hydrostatic extrusion [25]. The analytical model proposed in [25] is applicable for both hydrostatic extrusion and wire drawing processes and provides a good first approximation for this behavioral evaluation. Simulation details are presented in the following section. It must be noted that all the above simulations were merely intended to test the assumptions made earlier regarding the role of transverse and residual stresses and no verification of the experimental results was planned. Indeed, the exact development of residual stresses in the architectured composite wires is not straightforward. The simulations were applied towards identifying the potential source of the strengthening effect observed in the architectured Cu-Al wires (reported by [17]).

### 3.2. Numerical Modelling Details

The FEA software Abaqus/CAE (ABAQUS Inc., Johnston, RI, USA) was utilized to perform all simulations. All CCA samples were meshed using a mixture of hexahedral elements of type ‘C3D8R’ and wedge elements of type “C3D6′ (both of linear geometric order, from the standard element library) to generate a regular symmetric mesh. However, the ACCA sample was meshed using only hexahedral elements. Independent Al and Cu parts were then assembled by merging the interfacial elements that satisfies the perfect interface assumption and allows the development of transverse stresses. All models were assigned a boundary condition of type “ZSYMM” (symmetry about a constant z-plane) on the fixed end.

An arbitrary displacement of 0.005 mm (0.05% strain—within the reasonable range of elastic domain) was applied on all the CCA elastic models listed in Table 1. Engineering stress-strain data from tensile testing of the as-drawn pure Al and pure Cu samples were calibrated and converted into true stress-strain curves in Abaqus/CAE as input for elastoplastic simulation of the 3-mm diameter CCA samples mentioned in the previous section.

The elastic-plastic behavior of the aforementioned ACCA sample (≈25% Al) of a gauge length of 25 mm was also studied by straining it up to one percent. The use of CCA input for simulating the tensile testing of ACCA is acceptable to a fairly good approximation due to stress saturation in both Al and Cu at high strains (see [17]). Chinh et al. [26] also report stress saturation in highly strained Al.

In order to model the elastic-plastic behavior of CCA and ACCA composite wires in presence of residual stresses, the 25%Al-ACCA wire and its corresponding CCA sample (containing 25% Al) were chosen. Next, for comparison purposes, a cylindrical section of the same diameter of 1.5 mm was defined in the center of both ACCA and CCA samples. As explained in the previous section, residual stress values for simulation were taken from Ref. [25]. Therefore, predefined stress fields of −90 MPa (compressive) in the central cylinder and +10 MPa (tensile) in the remaining hollow cylinder were defined.

It should be noted that the aforementioned values were considered as single uniform values through the cross section of the wire rather than the actual curved-shape residual stress distributions (see the references presented in Section 3.1.2 Residual Stresses). This analytical model-based assumption was made for the sake of simplicity and comparison and does not satisfy the residuals stresses’ self-equilibrium requirement. It is however consistent with the literature in terms of the sign of expected residual stresses. Furthermore, to emphasize the favorable impact of compressive residual stresses in the central section of ACCA samples and to reveal its implication for the research problem, a separate simulation with −120 MPs (rather than −90 MPa) and +10 MPa was performed.

## 4. Results

### 4.1. Experimental Tensile Tests

**Figure 5:**
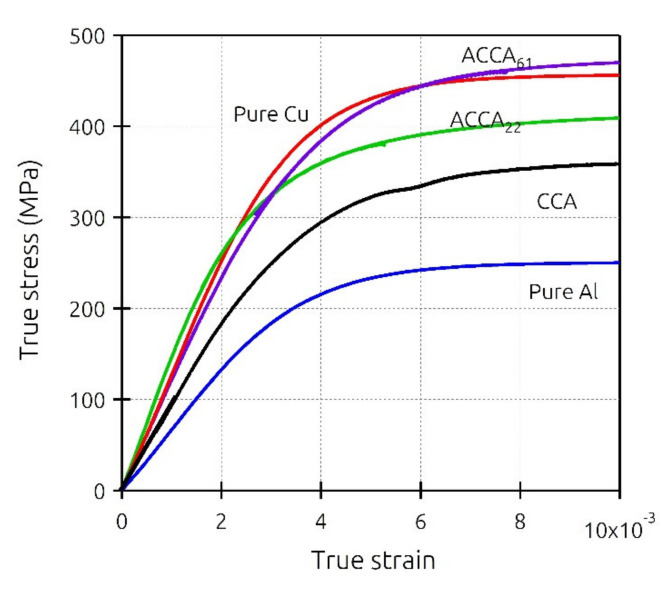
Illustration of the tensile curves of the different wires considered in this study: pure Al, pure Cu, copper clad aluminum (CCA) and architectured copper clad aluminum wire with 61 (ACCA_61_) and 22 restacked wires (ACCA_22_).

Figure 5 illustrates the mechanical behavior in tension of the earlier-mentioned CCA, ACCA_61_, and ACCA_22_ wires along with the pure copper and pure aluminum counterparts. When it comes to the CCA wire, the tensile curve lies between the pure Cu and pure Al ones, in good agreement with the rules of mixtures’ prediction. For a total strain of 0.01, the flow stresses of pure Cu and Al wires are about 450 and 250 MPa, respectively. For the same total strain, the CCA wire containing 50% Al exhibits a flow stress of about 360 MPa which is close to the rule of mixtures’ predicted value of 350 MPa.

Compared to the CCA case, the two ACCA wires show an increased flow stress that is closer (ACCA_22_) or even larger (ACCA_61_) than that of the corresponding pure copper wire. In that case, the rule of mixture is clearly not fulfilled revealing a complex mechanical behavior that can be attributed to the aforementioned transverse interactions or residual stresses.

### 4.2. CCA Elastic Simulations

In order to investigate the role played by the elastic-domain transverse interactions on the mechanical behavior of CCA an ACCA wires, elastic simulations were performed as a first attempt. When it comes to the CCA, the following graphs show the impact of the two parameters Young’s modulus and Al/Cu volume fraction on the development of tensile testing-induced radial and circumferential stresses versus the normalized distance along the diameter of each wire in the elastic domain.

**Figure 6:**
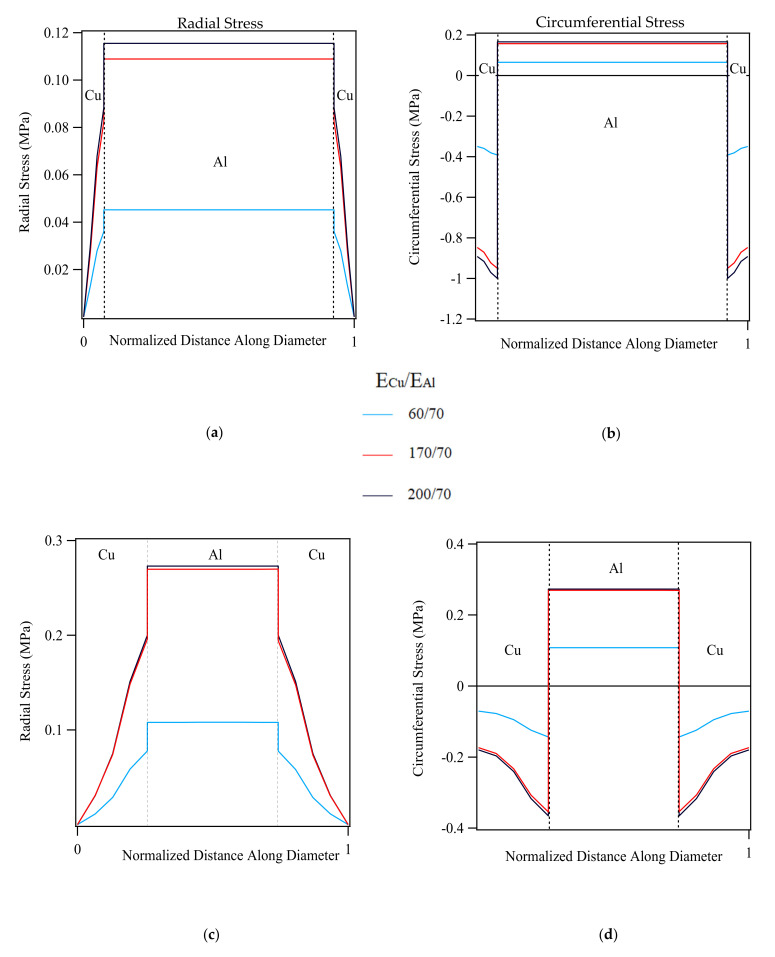
Effect of Young’s modulus and Al/Cu volume fraction on the magnitude and distribution of radial and circumferential stresses (**a**,**b**) 75%Al-CCA wire (**c**,**d**) 25%Al-CCA wire.

Effect of the two parameters on the distribution and magnitude of transverse stresses are visualized in Figure 6a–d, which represent the radial and circumferential stress profiles of the 75% Al-CCA sample and Figure 6c,d that illustrate those of the 25% Al-CCA sample. For a total elastic strain of 0.05%, average axial stress values of ≈63 and ≈45 MPa developed along the wire axis in the 25% Al- and 75% Al-sample, respectively. The ratio on the graphs’ legend is the ratio of the Young’s modulus of Cu to Al. The Al core and Cu case areas are delineated on the curves.

As observed in Figure 6, the magnitude of transverse stresses evolved in both 25%- and 75%Al-samples is utterly small (on the order of tenths of a megapascal). The magnitude of the corresponding axial stresses are, however, significantly higher as mentioned above. The magnitude of radial and circumferential stresses in both CCA samples of different volume fractions slightly increases as the Young’s modulus ratio becomes greater. It reaches its maximum for the ratio E_Cu_/E_Al_ = 200/70. Additionally, the higher the volume fraction of copper is, the greater the radial stress component in the Al core and Cu case would be. The circumferential stress component though increases in the Al core and decreases in the Cu case at higher volume fractions of Cu.

### 4.3. CCA and ACCA Elastoplastic Simulations

**Figure 7:**
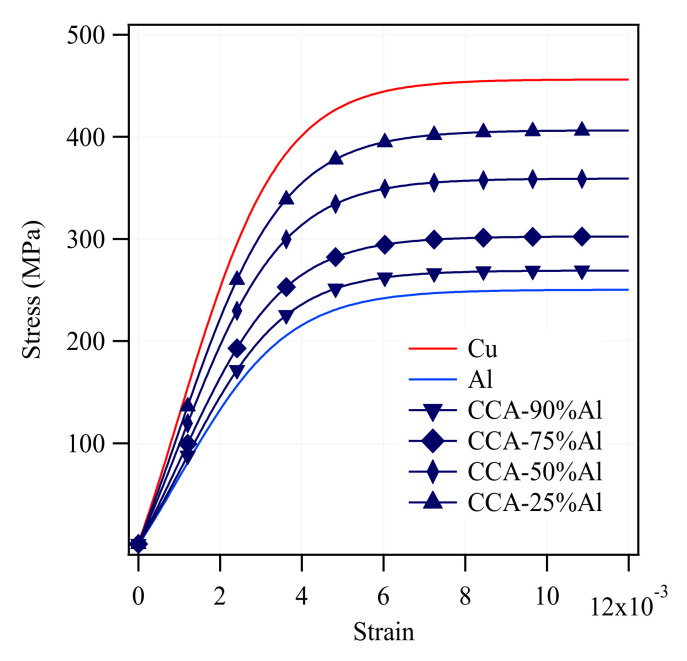
Simulation stress-strain curves of CCA samples of four different volume fractions.

**Figure 8:**
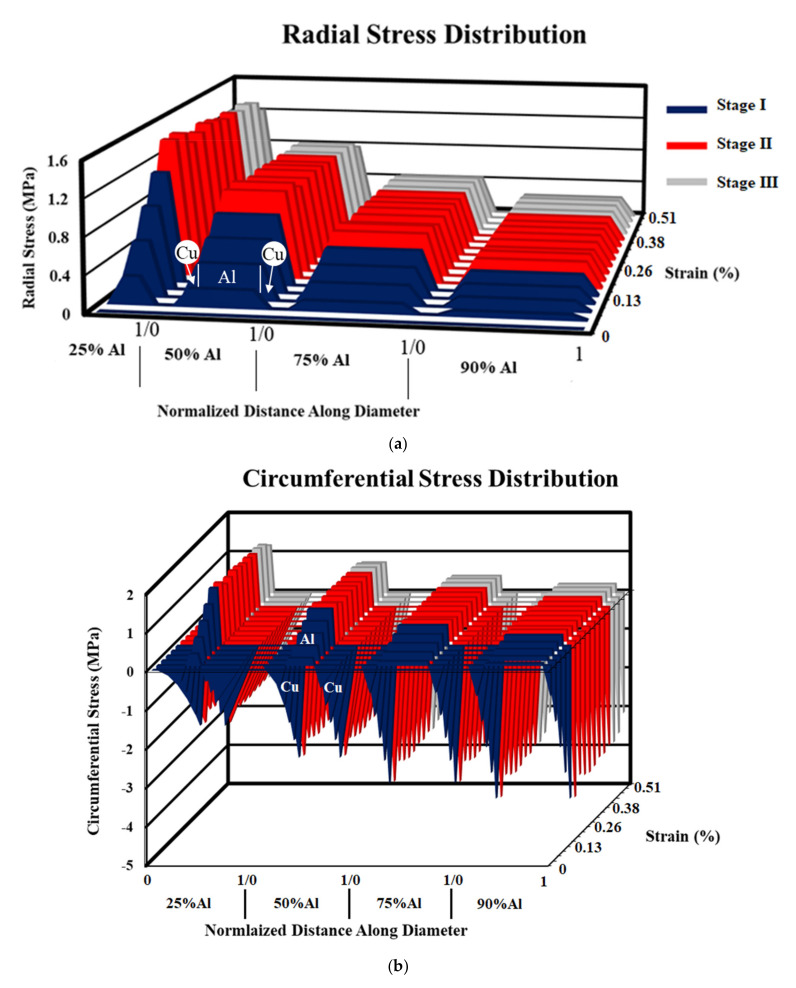
Development of (**a**) radial and (**b**) circumferential stresses in numerically tensile-tested CCA wires with various volume fractions.

Figure 7 shows the axial stress-strain curves of the CCA samples of the four aforementioned volume fractions simulated with elastic-plastic behavior along with the experimental pure Cu and Al curves. The tensile stress increases with a rise in the Cu volume fraction as expected. Figure 8a,b summarize how transverse stresses evolve during numerical tensile testing of CCA wires with four different volume fractions. The 3D graphs of Figure 8 contain two horizontal and one vertical axes. One of the two horizontal axes represents the axial strain and the other axes show the distribution of radial/circumferential stress (at each strain level) versus the normalized distance along the diameter of each wire between each 0 and 1 with the corresponding volume fraction of Al determined. The Al core and Cu case areas are depicted on the distribution profiles. The three stages indicated in three different colors correspond to the strain ranges of the three common regions on the axial stress-strain curve of concentric composite cylinders (CCA wires in this study) arising from the varying Poisson’s ratio of each phase during tensile testing [9]. The main purpose of the 3D diagrams is to demonstrate the order of magnitude of transverse stresses that develop during numerical tensile testing of CCA wires and therefore a further explanation about those three regions is avoided. The maximum magnitude of radial and circumferential stresses in samples of all volume fractions is reached at the onset of the second stage as the first component (Cu) begins yielding. A comparison between the magnitude of the various stress tensor components from Figure 7 and Figure 8 underpins the fact that the axial stress remains by far the predominant component in both elastic and plastic domains of CCA wires during tensile-testing.

**Figure 9:**
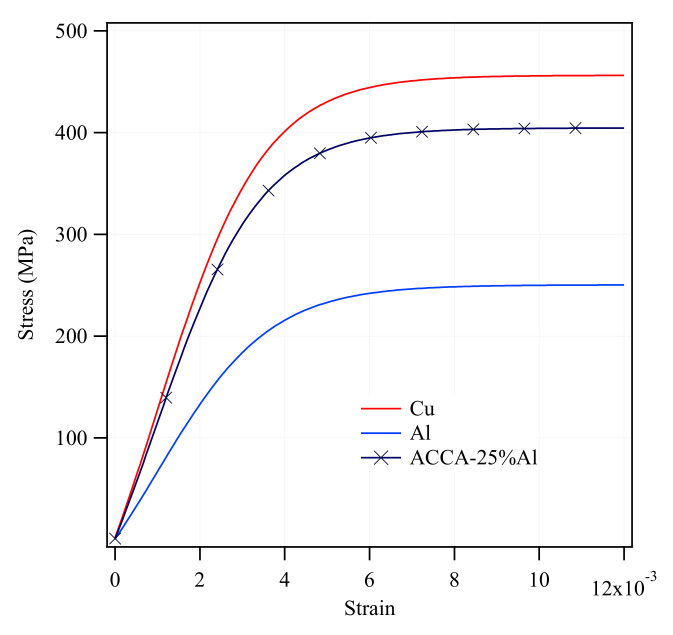
Simulation stress-strain curve of ≈25%Al-ACCA sample.

**Figure 10:**
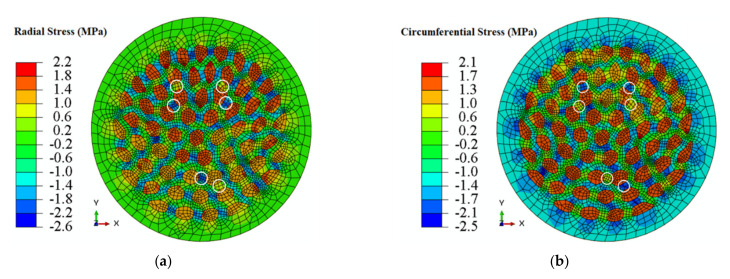

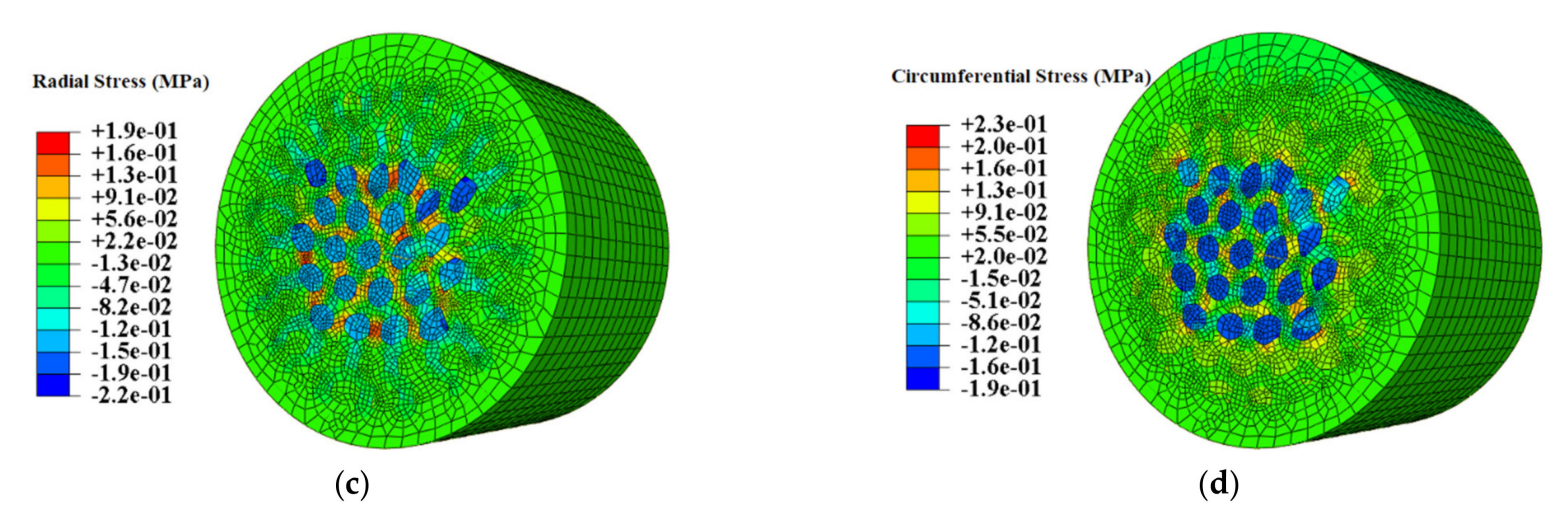
(**a**) Radial and (**b**) circumferential stress distribution and magnitude of ≈25%Al-ACCA sample (at ≈0.2% strain) (**c**) radial and (**d**) circumferential stress distributino at the end of the numerical tensile test (at 1% strain).

Figure 9 shows the simulation axial-stress-strain curve of the ACCA wire containing 25% Al. Radial and circumferential stress fields at a total strain of ≈0.2% are illustrated in Figure 10a,b, respectively. This is the strain level at which the maximum magnitude of transverse stresses was reached during numerical tensile-testing of the architectured sample. Similar to CCA wires, the above strain level corresponds to the onset of stage II at which one of the components begins yielding first in the ACCA sample. The radial and circumferential stress distribution patterns across the ACCA wire cross-section is though distinctively different from those of the CCA wires throughout tensile testing. The most prominent feature is the channels of negative and positive transverse stresses evolving in the inter-fiber space of the copper matrix, pairs of which are depicted in Figure 10a,b (white circles). Figure 10c,d show the distribution of radial and circumferential stresses at the end of the numerical tensile test (at ≈1% strain). The magnitude of transverse stresses nears zero and their distribution becomes homogeneous at this stage. Note that a coarser mesh than that of Figure 3b was used to reduce the computational cost since the numerical solution was well converged with even coarser mesh.

### 4.4. Residual Stresses

**Figure 11:**
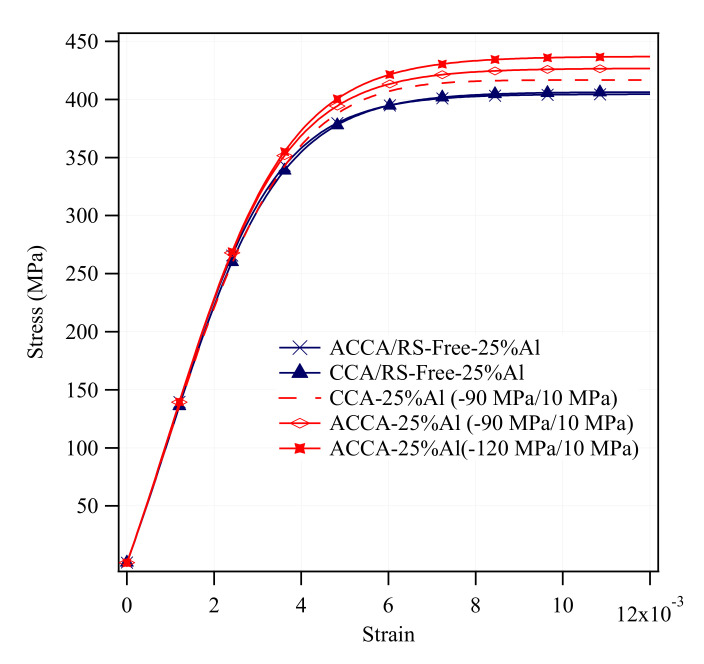
Numerical stress-strain curves of ≈25%Al-ACCA and 25%Al-CCA wires (with and without residual stress fields).

Stress-strain curves of numerically tensile-tested ≈25%Al-ACCA and 25%Al-CCA wires, with and without predefined residual stress fields, are plotted in Figure 11. The stress-strain curves of residual stress-free ACCA and CCA lie over one another as shown in this graph. Figure 11 allows comparisons to be made between CCA and ACCA samples. It reveals the role of architecture. It is implicative of the consequential impact of the residual stress profile and particularly compressive residual stresses built up in the inner section of cold-drawn samples. According to Figure 11, −90 MPa of compressive and 10 MPa of tensile residual stress with the earlier-mentioned configuration put the yield strength of CCA and ACCA by about 10 and 15 MPa above the stress-free curves respectively. A higher-magnitude compressive residual stress of −120 MPa (i.e., −120 MPa/10 MPa) increases the yield strength by about 20 MPa. Please note that these positive deviations are not meant to imply that the presence of residual stresses improve the yield strength. Near-surface tensile residual stresses could actually have deleterious effects on the tensile strength as referred to earlier. It is merely because of the way the residual stress fields are defined based on the analytical model in [25]. Residual stress-free curves are simply presented as a baseline for comparison. The red curves with residual stress fields are to be compared.

## 5. Discussion and Outlook

The experimental results revealed a slightly increase in the tensile flow stress of the two architectured Cu-Al wires compared to the rule of mixtures’ prediction. The two key parameters I- transverse stresses and II- processing-induced residual stresses were investigated via finite element analysis as the potential sources of this behavior. The two different features and their implications on the mechanical behavior are discussed in the following section.

### 5.1. Elastic-Domain Transverse Stresses in CCA Samples

The features of interest in the elastic-domain simulations of CCA wires were the order of magnitude of radial and circumferential stresses and the ways this magnitude changes influenced by the parameters involved. Figure 6a–d illustrate how the two parameters Young’s modulus and volume fraction of each phase affect the evolution of transverse stresses as explained in the results section. It is evident from those figures that the maximum magnitude of both radial and circumferential stress components is on the order of tenths of a megapascal in all cases. This is while the axial stress component developed in the CCA samples for a corresponding elastic strain of 0.05% (from the linear rule of mixtures) is on the order of about 63 MPa for the 25%Al-ACCA and 45 MPa for the 75%Al-ACCA sample. This implies the quite weak contribution of transverse stresses evolved in the elastic domain of axially strained CCA wires, consistent with the analytical model developed by Ebert et al. [9] for concentric cylindrical composites.

### 5.2. Transverse Stresses in CCA and ACCA Samples with Elastic-Plastic Behavior

It was pointed out earlier that there is a strain range between the onset of plasticity in the first and second components of a bimetallic cylindrical composite during which a greater Poisson’s ratio difference and consequently higher-magnitude transverse stresses may be expected. However, two other major factors also determine the significance of the developed radial and circumferential stresses contributing to the axial stress-strain behavior. The two other factors are 1- volume fraction of each phase, 2- the ratio of their elastic moduli [9]. The Young’s moduli of experimentally tensile-tested as-drawn pure copper and pure aluminum are 129 and 66 GPa, respectively (Figure 2). Figure 8a,b with four different volume fractions of numerically tensile-tested CCA wires provide a good approximation of the order of magnitude of radial and circumferential stresses. It can easily be seen from these figures that the maximum magnitude of transverse stresses would not exceed a few megapascals for different Al/Cu volume fractions. This is because of the relatively small ratio of the Young’s modulus of Cu to that of Al (calculated from the experimental stress-strain curves) and again implies the negligible contribution of transverse stresses to the axial stress-stain behavior of CCA wires whose axial stress-strain curves are plotted in Figure 7. Furthermore, it can be deduced from Figure 8b that the greater the volume fraction of one component is, the smaller the magnitude of circumferential stress would be in that component.

Evolution of the maximum radial and circumferential stress values during numerical tensile-testing of a ≈25%Al-ACCA wire modelled from its actual transverse cross-section (see Figure 3a,b) is shown in Figure 10a,b. The maximum magnitude of transverse stresses (about ±2 MPa) developed in the ACCA sample is of almost the same order of magnitude of maximum transverse stresses in its CCA counterpart (25%Al–75%Cu). This indicates the fact that architecture does not change the magnitude of transverse stress components and the magnitude is merely a function of volume fraction. The distribution of radial and circumferential stresses, however, interestingly changes due to the novel fiber-matrix configuration of ACCA compared to CCA. It can be observed in Figure 8a that the sign of the radial stress component in both Al core and copper case of CCA wires remains positive throughout the tensile test. Figure 8b also indicates that the sign of the circumferential component in CCA wires is positive in the Al core and negative in the Cu case all along the test.

Nevertheless, there are channels of both negative and positive radial and circumferential stresses in the inter-fiber space of the Cu matrix of ACCA wires throughout the tensile test, as shown in Figure 10a,b. This feature may have important implications in terms of interfacial damage initiation and propagation.

However, the feature of interest in this study is the magnitude of transverse stresses developed during tensile testing of CCA and ACCA wires. To conclude Section 5.1 and Section 5.2, it can be inferred that the magnitude of radial and circumferential stresses evolved in CCA and ACCA wires is quite small that transverse stresses cannot be considered as the underlying reason behind the improved yield strength of ACCA wires.

### 5.3. Mechanical Bonding at the Al-Cu Interface

The mechanical bonding at the Al-Cu interface of both cold-drawn CCA and ACCA wires is one of the key aspects to be studied when it comes to the axial stress-strain behavior of these bimetallic composites. The focus of this numerical study is, however, to discover the origin of the enhanced strength of ACCA. There are studies attributing the positive deviation from the rule of mixtures (RoM) and improved strength of similar bimetallic composite systems, such as Cu-Nb, to the interface. Those observed strengthening effects have been justified by models such as Hall-Petch Barrier and Geometrically Necessary Dislocations (GND). However, both models are valid where there is a size effect involved and interface (fiber) spacing is on the order of nanometer [27]. Whilst there are nearly 200 grains, as large as 500 nm each, situated in the space between every two Al fibers in the 25%Al-ACCA sample investigated in this study and therefore no size effect is expected. Perfect interface was one of the assumptions made in this work given that any sort of imperfection can potentially bring about loci of stress concentration and be detrimental to the yield strength. Although, all the interface-related discussions are relevant as long as the fiber-matrix bonding is in place. Possible sources of strengthening are the focal points of the current investigation and therefore the Al-Cu interface was not considered since it is not expected to bring about any strengthening effect as argued above. Although, it is intended to conduct a separate study into understanding the bond strength and interfacial behavior of Al-Cu composite wires in both as-drawn and heat-treated conditions in prospect.

### 5.4. Residual Stresses

A comparison-based approach was adopted towards realizing the impact of residual stresses on the axial stress-strain behavior of CCA and ACCA wires. One should note that residual stresses already contribute to the tensile stress-strain curve of the cold-drawn pure Cu and Al rod samples used as simulation input. However, co-deformation and architecture are expected to form more compressive residual stresses consistent with the following analysis. As illustrated in Figure 11, the simulation curves of the residual stress-free 25%Al-ACCA and 25%Al-CCA samples (dark blue curves) almost entirely overlap because of their similar Al volume fraction as discussed in the Section 5.2. In a first attempt to discover the net effect of architecture in presence of predefined residual stress fields, CCA and ACCA samples were compared irrespective of the different amount of plastic deformation they actually experience.

A comparison between the numerical stress-strain curves of the 25%Al-ACCA and 25%Al-CCA wires in Figure 11 shows that the ACCA curve (red with horizontal diamond markers) lies well above the CCA curve (dashed red curve). This clearly demonstrates that the architecture can improve the yield strength under entirely similar conditions (identical residual stress field configurations—see Figure 4) in presence of drawing-induced residual stresses. This can be ascribed to the fact that the novel fiber-matrix configuration of ACCA compared to that of CCA of the same volume fraction, brings more of the stronger phase (that is the copper matrix) into the central part of the composite wire where there is a region of processing-induced compressive residual stresses. This mechanism is consistent with the smaller deviation of the ACCA_22_ wire from the rule of mixtures’ prediction when compared with the ACCA_61_ since the volume fraction of copper in the compressive stress area is lower in ACCA_22_.

Moreover, a second comparison with the purpose to provide insights into discovering the origin of the improved strength of ACCA can be made between the two similar ACCA_61_ simulations with compressive residual stress fields of different magnitude (solid red curves with markers—see Figure 9). The stress-strain curve of the ACCA sample with a greater compressive residual stress field obviously deviates upwards and shows greater yield strength by lying above. To determine the implications, as mentioned in the Residual Stresses subsection of the Numerical Procedure section, drawing-induced residual stresses come from the non-uniform plastic deformation evolved during the process according to the literature. Bringing some portion of the copper to the center of the wire in the architectured samples could be expected to bring about deformation that is more homogeneous. This can reduce the undesirable tensile residual stresses near the surface of the wire that in turn leads to the prevalence of compressive residual stresses built up in the central region of ACCA wires. Hence, the stress-strain curve of an ACCA sample can exhibit significantly high yield strength in the exact same fashion that the ACCA sample with a larger compressive residual stress field behaves in Figure 11.

This strong implication necessitates further simulations and experimental work to model the manufacture process and drawing-induced residual stresses along with experimental measurements of these stresses. A sound comprehension of the tensile behavior of Al-Cu composite wires lays the groundwork for developing a deeper understanding of the mechanical properties of both conventional and novel configurations with different heat-treatment conditions, which in turn leads to optimum production of theses wires.

## 6. Conclusions

The tensile behavior of as-drawn conventional copper clad aluminum and architectured Al-Cu composite wires reveals an improvement in the strength of the architectured fiber-matrix configuration. The influence of the two key parameters 1-transverse and 2-residual stresses as the potential sources of the above behavior were examined using finite element analysis. The tensile response of axially strained conventional (CCA) and architectured (ACCA) copper-clad aluminum wires were then simulated under the influence of those two parameters. The findings suggest the following conclusions:

- The effect of the various possible Al-Cu Young’s modulus ratios and volume fractions on the evolution and magnitude of transverse stresses was found to be trivial (a few tenths of a megapascal) in Al-Cu composite wires.
- Contribution of transverse stresses to the axial stress-strain behavior of both CCA and ACCA wires is insignificant (3 MPa on average at most).
- Distribution of transverse stresses in architectured Al-Cu wires is interestingly different from that of conventional CCA wires showing channels of both negative and positive radial and circumferential stress components throughout the tensile test.
- Drawing-induced residual stresses with magnitudes on the order of tens/hundreds of megapascals have strong implications in terms of the observed strengthening effect of architecture.
- ACCA wires show improved strength compared to CCA wires in presence of identical compressive and tensile residual stress fields because of the novel fiber-matrix configuration of the architectured samples.
- More uniform plastic deformation in ACCA wires and the formation of compressive residual stresses in the central portion of the Cu matrix are highly likely the reasons behind the enhanced yield strength observed in ACCA composite wires.

## Figures and Tables

**Table 1:** Elastic simulation parameters for investigating the development of transverse stresses in CCA wires.

Volume Fractions	75%Al–25%Al	
Phase	Poisson’s Ratio	Young’s Modulus (GPa)
Cu	0.31	60–170–200
Al	0.33	70

**Table 2:** Elastoplastic simulation parameters for investigating the development of transverse stresses in CCA wires.

Volume Fractions	25% Al–50% Al–75% Al–90% Al	
Phase	Poisson’s Ratio	Young’s Modulus (GPa)
Cu	0.31	≈129
Al	0.33	≈66

## Data Availability

The raw/processed data required to reproduce these findings cannot be shared at this time as the data also forms part of an ongoing study.
